# INCLUSIVE MENTORSHIP IN GEROPSYCHOLOGY: CULTIVATING SAFETY, TRUST, HUMILITY, AUTHENTICITY, AND COMMUNICATION

**DOI:** 10.1093/geroni/igae098.0316

**Published:** 2024-12-31

**Authors:** Ira Yenko, Flora Ma, Anna Blanken, Claudia Son, Laurie Chin

**Affiliations:** Stanford University School of Medicine, Stanford, California, United States; Stanford University School of Medicine, Stanford, California, United States; San Francisco VA Healthcare System, San Francisco, California, United States; Biola University, La Mirada, California, United States; University of Indianapolis, Indianapolis, Indiana, United States

## Abstract

Attention to equity, diversity, and inclusion (EDI) in mentorship is paramount; as such, the Society of Clinical Geropsychology’s Mentorship and Diversity Committees have joined forces to outline concrete methods to improve such practices. Mentoring across differences can promote intercultural mentoring relationships that embrace diversity and support inclusion. This presentation explores the significance of EDI and offers practical strategies to shift towards a more inclusive perspective in mentorship, with special attention to the development of geropsychology trainees. This includes recognizing and valuing the unique perspectives, experiences, and identities that both mentors and mentees bring to the table. By fostering an inclusive mentorship environment, we can maximize opportunities for individuals with marginalized identities and ensure equal access to resources, support, and opportunities. This presentation will review the following strategies: (1) Creating a safe and welcoming space for mentees and maximizing trainee comfort in expressing their thoughts, concerns, and questions; (2) Being reliable, consistent, and supportive by establishing a mentor-mentee agreement outlining expectations, goals, and boundaries to set the stage for a trusting relationship; (3) Authenticity and acknowledging there is no one “right” way to be a geropsychologist; (4) Humility to create a culture of feedback and growth and remain open to diverse perspectives; (5) Actively listening to demonstrate empathy and validate mentee experiences; (6) Encouragement. By fostering a culture of mentorship to uplift, support, and empower the next generation, we pave the way for a future where geriatric mental health is inclusive and responsive to the diverse needs of older adults.

